# Plants as Biofactories: Postharvest Stress-Induced Accumulation of Phenolic Compounds and Glucosinolates in Broccoli Subjected to Wounding Stress and Exogenous Phytohormones

**DOI:** 10.3389/fpls.2016.00045

**Published:** 2016-02-10

**Authors:** Daniel Villarreal-García, Vimal Nair, Luis Cisneros-Zevallos, Daniel A. Jacobo-Velázquez

**Affiliations:** ^1^Centro de Biotecnología-FEMSA Tecnológico de Monterrey – Campus MonterreyMonterrey, Mexico; ^2^Department of Horticultural Sciences, Texas A&M University, College StationTX, USA

**Keywords:** broccoli, glucosinolates, phenolic compounds, wounding stress, ethylene, methyl jasmonate, neoglucobrassicin

## Abstract

Broccoli contains high levels of bioactive molecules and is considered a functional food. In this study, postharvest treatments to enhance the concentration of glucosinolates and phenolic compounds were evaluated. Broccoli whole heads were wounded to obtain florets and wounded florets (florets cut into four even pieces) and stored for 24 h at 20 °C with or without exogenous ethylene (ET, 1000 ppm) or methyl jasmonate (MeJA, 250 ppm). Whole heads were used as a control for wounding treatments. Regarding glucosinolate accumulation, ET selectively induced the 4-hydroxylation of glucobrassicin in whole heads, resulting in ∼223% higher 4-hydroxyglucobrassicin than time 0 h samples. Additionally, glucoraphanin was increased by ∼53% in whole heads treated with ET, while neoglucobrassicin was greatly accumulated in wounded florets treated with ET or MeJA, showing increases of ∼193 and ∼286%, respectively. On the other hand, although only whole heads stored without phytohormones showed higher concentrations of phenolic compounds, which was reflected in ∼33, ∼30, and ∼46% higher levels of 1,2,2-trisinapoylgentiobose, 1,2-diferulolylgentiobiose, and 1,2-disinapoyl-2-ferulolylgentiobiose, respectively; broccoli florets stored under air control conditions showed enhanced concentrations of 3-*O*-caffeoylquinic acid, 1,2-disinapoylgentiobiose, and 1,2-disinapoyl-2-ferulolylgentiobiose (∼22, ∼185, and ∼65% more, respectively). Furthermore, exogenous ET and MeJA impeded individual phenolics accumulation. Results allowed the elucidation of simple and effective postharvest treatment to enhance the content of individual glucosinolates and phenolic compounds in broccoli. The stressed-broccoli tissue could be subjected to downstream processing in order to extract and purify bioactive molecules with applications in the dietary supplements, agrochemical and cosmetics markets.

## Introduction

Broccoli (*Brassica oleracea* L. var. *Italica*) is a very important crop in economic terms. According to the Food and Agricultural Organization of the United Nations statistical database (FAOSTAT), in the year 2013 ∼22 million tons of broccoli and cauliflowers were produced worldwide. Broccoli production and consumption per capita has greatly increased over the last two decades. From 1993, broccoli worldwide production augmented by ∼120% (Food and Agricultural Organization of the United Nations [FAOSTAT], n.d.), whereas broccoli consumption per capita increased by ∼50% in the United States (Economics, Statistics, and Market Information System [ERS], n.d.). The increased economic importance of broccoli is in part due to an increase in the number of consumers interested in eating more functional foods (Agricultural Marketing Resource Center [AgMRC], n.d.).

Broccoli contains high levels of phenolics and glucosinolates, which are among the most effective bioactive molecules that prevent chronic and degenerative diseases (Vinson et al., 1998; Fahey and Talalay, 1999; Heo et al., 2007; Rodríguez-Cantú et al., 2011). Phenolic compounds are widely known as potent antioxidants (Rice-Evans et al., 1997; Jacobo-Velázquez and Cisneros-Zevallos, 2009; Brewer, 2011; Del Rio et al., 2013). Likewise, glucosinolates are amino acid-derived secondary metabolites that when hydrolyzed by a β-thioglucosidase (myrosinase) yield isothiocyanates (Radojčić-Redovniković et al., 2008), which are strong inducers of phase II enzymes, helping to prevent oxidative stress caused by reactive electrophile species (Fahey and Talalay, 1999). Besides, sulforaphane, one of the most common isothiocyanates in broccoli, has been shown to eradicate infections by *Helicobacter pylori* (Fahey et al., 2002) and inhibit chronic inflammatory processes (Juurlink, 2001).

It has been reported that the application of postharvest abiotic stresses (i.e., wounding, UV-light radiation, and exogenous phytohormones) induce the accumulation of health-promoting compounds in plants (Cisneros-Zevallos, 2003). In addition, when horticultural crops are subjected to extreme postharvest abiotic stress conditions, the genetic potential of plants to produce secondary metabolites can be exploited inducing the accumulation of high levels of bioactive molecules (Jacobo-Velázquez and Cisneros-Zevallos, 2012). In the particular case of broccoli, the application of certain postharvest abiotic stress conditions could be used as an approach to induce the activation of phenolic and glucosinolate biosynthesis pathways, leading to an enhancement of its nutraceutical content. This becomes particularly relevant when alternative uses for horticultural crops are needed; especially in fresh produce not meeting quality standards for human consumption, which represent one third of worldwide production (Food and Agriculture Organization of the United Nations [FAO], n.d.).

Plant hormones such as methyl jasmonate (MeJA) and ethylene (ET) have been used as elicitors of high-value antioxidants in several plant models. MeJA, a phytohormone involved in diverse developmental processes and plant defense mechanisms, has been studied as a pre-harvest elicitor to enrich glucosinolate and/or phenolic content in *Brassica rapa* and broccoli (Liang et al., 2006; Kim and Juvik, 2011; Ku et al., 2014; Liu et al., 2014). Moreover, ET has also been shown to be effective in the activation of glucosinolate biosynthetic genes in *Arabidopsis* (Mikkelsen et al., 2003). On the other hand, the accumulation of phenolic compounds in fresh produce in response to postharvest treatment with ET or MeJA has been previously reported (Heredia and Cisneros-Zevallos, 2009a). In the specific case of broccoli, the pre-harvest application of MeJA has been shown to increase the concentration of total glucosinolates in broccoli florets (Ku et al., 2013a,b). Additionally, previous reports have shown that MeJA may induce the accumulation of total phenolics and glucosinolates in broccoli sprouts (Pérez-Balibrea et al., 2011). However, the effect of postharvest treatments with exogenous ET and MeJA on the accumulation of glucosinolates and phenolic compounds in broccoli has not been reported.

Recently, it was reported that wounding triggers the production of reactive oxygen species (ROS), which induce the activation of primary and secondary metabolism in plants (Jacobo-Velázquez et al., 2015). Furthermore, the authors reported that other signaling molecules, such as ET and jasmonic acid (JA), which are produced after wounding, play key roles as ROS levels modulators, and mediate the expression of secondary metabolism genes, triggering the accumulation of specific secondary metabolites. Therefore, the accumulation of antioxidants in plants has also been studied as an effect of wounding during postharvest (Reyes and Cisneros-Zevallos, 2003; Jacobo-Velázquez et al., 2011; Surjadinata and Cisneros-Zevallos, 2012; Torres-Contreras et al., 2014a,b). In the case of broccoli, it has been reported that wounding triggers the biosynthesis and accumulation of indolic glucosinolates (Verkerk et al., 2001). Abiotic stress has been previously evaluated as a strategy to enhance the nutritional content of broccoli samples. However, the study of postharvest treatments has been scarce and the effect of combined application of two or more postharvest abiotic stresses has not been thoroughly evaluated although previous reports have shown that the *de novo* biosynthesis of glucosinolates is more likely to occur during postharvest storage (Ku et al., 2013a).

Wounding has been proven to be one of the most effective postharvest abiotic stresses for the activation of the phenylpropanoid metabolic pathway in plants (Reyes and Cisneros-Zevallos, 2003; Surjadinata and Cisneros-Zevallos, 2012; Torres-Contreras et al., 2014a). In addition, the application of ET and MeJA in wounded broccoli samples and their effect on the accumulation of phenolic compounds and glucosinolates has not been evaluated yet. Therefore, the objective of this research was to evaluate the effect of wounding stress alone and in combination with exogenous ET or MeJA, on the accumulation of total and individual phenolic compounds and glucosinolates during storage (24 h at 20°C) of broccoli tissue. Although it is well known that ET induces chlorophylls degradation in broccoli (Tian et al., 1994), in the present study quality parameters were not considered relevant to evaluate, since the objective was to find alternative uses to broccoli not intended for human consumption, such as biofactory of high value phenolic and glucosinolate compounds. The stressed-broccoli tissue could be subjected to downstream processing in order to extract and purify bioactive molecules with applications in the dietary supplements, agrochemical and cosmetics markets.

## Materials and Methods

### Chemicals

Sulfatase from *Helix pomatia*, sinigrin hydrate, sephadex A-25, 3-*O*-caffeoylquinic acid (3*-O*-CQA*)*, acetonitrile (HPLC grade), methanol (HPLC grade), sodium acetate, MeJA, and orthophosphoric acid were obtained from Sigma Chemical Co. (St. Louis, MO, USA). ET was purchased from Infra (Naucalpan, MEX, Mexico). Acetic acid was purchased from Desarrollo de Especialidades Quimicas (Monterrey, NL, Mexico). Desulfoglucoraphanin was obtained from Santa Cruz Biotechnology (Dallas, TX, USA).

### Plant Material, Processing, and Storage Studies

Broccoli (*Brassica oleracea*) was obtained on June 2014, once from a local market (HEB, Monterrey, Mexico), washed, and disinfected with chlorinated water (200 ppm, pH 6.5). All samples were supplied by the same grower. Whole heads were used as control samples for wounding stress. Florets and wounded florets (cut into four even pieces with a commercial straight-edged knife) were used as wounding treatments. Wounded and whole samples were stored inside hermetic plastic containers with periodic ventilation to avoid CO_2_ accumulation over 0.5% (v/v). One broccoli head was used per replica of each treatment. Three biological replicates were performed for each treatment.

Ethylene and MeJA were applied as reported by Heredia and Cisneros-Zevallos (2009a), where ET was directly injected into the plastic containers (1 mL/L) and MeJA was applied by wetting a Whatman No. 4 filter paper (Whatman Inc., Piscataway, NJ, USA) over a Petri dish (0.25 mL/L). All samples were stored for 24 h in an incubator (VWR, Radnor, PA, USA) at 20°C to determine the treatment that yields a maximum accumulation of phenolic compounds and glucosinolates. Collected samples were freeze-dried (Labconco, Kansas City, MO, USA) prior to extraction of phytochemicals. The concentration of individual phenolics and glucosinolates was determined before and after storage.

### Analysis of Phenolic Compounds by High-Performance Liquid Chromatography-Diode Array Detection (HPLC-DAD) and HPLC-Electrospray Ionization (ESI)-MS^n^

#### Extraction of Phenolic Compounds

For the chromatographic detection and quantification of individual phenolic compounds, a methanol extract was prepared. Broccoli powder (0.5 g) was homogenized with methanol (20 mL) using a tissuemizer (Advanced homogenizing system, VWR). Subsequently, homogenates were centrifuged (9000 x *g*, 1 h, 4°C). The clear supernatant was filtered using nylon membranes (0.45 μm, VWR) prior to injection to the chromatographic systems.

#### Analysis of Phenolic Compounds by HPLC-DAD and HPLC-ESI-MS^n^

The identification and quantification of individual phenolic compounds were performed as described by Torres-Contreras et al. (2014a). Briefly, 10 μL of the extract were injected in the HPLC-DAD system (1260 Infinity, Agilent Technologies, Santa Clara, CA, USA). Separation was done on a 4.6 mm × 250 mm, 5 μm particle size, C18 reverse phase column (Luna, Phenomenex, Torrance, CA, USA). Mobile phases consisted of water (phase A) and methanol/water (60:40, v/v, phase B) both adjusted at pH 2.4 with orthophosphoric acid. The gradient solvent system was 0/100, 3/70, 8/50, 35/30, 40/20, 45/0, 50/0, and 60/100 (min/% phase A) at a constant flow rate of 0.8 mL/min. Phenolic compounds were detected at 320 nm. Chromatographic data of analyses was processed with OpenLAB CDS ChemStation software (Agilent Technologies, Santa Clara, CA, USA).

Mass spectra were obtained on a MS Finnigan LCQ Deca XP Max, Ion trap mass spectrophotometer coupled at the exit of the DAD and equipped with a *Z*-spray ESI source, and run by Xcalibur version 1.3 software (Thermo Finnigan, San Jose, CA, USA). Separations were conducted in a Phenomenex Synergi^TM^ 4 μm Hydro-RP 80 Å (2 mm × 150 mm) with a C18 guard column, and a flow rate of 200 L/min from the DAD eluent was directed to the ESI interface using a flow-splitter. Mobile phases were adjusted to pH 2.4 with formic acid. Nitrogen was used as desolvation gas at 275°C and a flow rate of 60 L/h, and helium was used as damping gas. ESI was performed in the negative ion mode using the following conditions: sheath gas (N_2_), 60 arbitrary units; spray voltage, 1.5 kV; capillary temperature, 285°C; capillary voltage, 45.7 V; tube lens offset, 30 V.

Individual phenolics were identified on the basis of retention time, UV spectra, and their mass-to-charge ratio as compared with authentic standards and a previous report (Vallejo et al., 2003). For the quantification of phenolic compounds, a standard curve of 3-*O*-CQA was prepared in the range of 0.5–100 μM. The concentration of phenolics was expressed as mg of 3-*O*-CQA equivalents per kg of broccoli dry weight (DW).

### Analysis of Glucosinolates by High-Performance Liquid Chromatography-Diode Array Detection (HPLC-DAD) and HPLC-Electrospray Ionization (ESI)-MS^n^

#### Extraction and Desulfation of Glucosinolates

For the chromatographic determination of glucosinolates, extraction, and desulfation was done as described by Kiddle et al. (2001) with modifications described by Saha et al. (2012). Briefly, 10 mL of methanol:water (70:30, v:v), previously heated for 10 min at 70°C, were added to broccoli powder (0.2 g) followed by 50 μL of a 3 mM solution of sinigrin as internal standard. Samples were vortexed and incubated at 70°C for 30 min to ensure myrosinase inactivation. The extracts were removed from the water bath, left to cool at room temperature and centrifuged (3000 × *g*, 5 min, 4°C).

Afterward, glucosinolates were desulfated and purified using disposable polypropylene columns (Thermo Fisher Scientific, Waltham, MA, USA). To prepare the columns, 0.5 mL of water were added, followed by 0.5 mL of prepared Sephadex A-25 and an additional 0.5 mL of water. Clear supernatant (3 mL) was added into a prepared column and was allowed to drip through slowly. Columns were washed with 2 × 0.5 mL of water followed by 2 × 0.5 mL of 0.02 M sodium acetate. Purified sulfatase (75 μL) was added to each sample and was left at room temperature overnight (12 h). Desulfoglucosinolates were eluted with a total of 1.25 mL of water (0.5 mL + 0.5 mL + 0.25 mL).

#### Analysis of Desulfoglucosinolates by HPLC-DAD and HPLC-ESI-MS^n^

Determination of desulfoglucosinolates was performed as reported by Vallejo et al. (2003) with slight modifications. Chromatographic separations were done on the same chromatographic systems and reverse phase columns used for the analysis of phenolic compounds. Separation of desulfoglucosinolates in the HPLC-DAD system was achieved using water (phase A) and acetonitrile (phase B) as mobile phases with a flow rate of 1.5 mL/min and a gradient of 0/100, 28/80, 30/100 (min/% phase A) with an injection volume of 20 μL. All compounds were detected at 227 nm.

For the HPLC-ESI-MS*^n^* analyses, the gradient of the solvent system used to obtain mass spectra was 0/99, 16/80, 18/10 (min/% phase A) and a flow rate of 350 μL/min. Nitrogen was used as desolvation gas at 275°C and a flow rate of 60 L/h, and helium was used as damping gas. ESI was performed in the negative ion mode using the following conditions: sheath gas (N_2_), 60 arbitrary units; spray voltage, 5 kV; capillary temperature, 285°C; capillary voltage, 48.5 V; tube lens offset, 30 V.

Individual glucosinolates were identified on the basis of retention time, UV spectra, and their mass-to-charge ratio as compared with authentic standards and previous reports (Hansen et al., 1996; Vallejo et al., 2003; Barbieri et al., 2008; Miglio et al., 2008). A standard curve of desulfoglucoraphanin was prepared in the range of 0–700 μM for the quantification of glucosinolates. The concentration of individual glucosinolates was expressed as mmol of desulfoglucoraphanin equivalents per kg of broccoli.

### Statistical Analysis

Replication was achieved by repeating treatment under the same conditions. All treatments were run concurrently. All reported data were pooled from repeated independent treatment. There were three replicates per treatment (*n* = 3). Statistical analyses were performed using the three replicates. Data represent the mean values of samples and their standard error. Analyses of variance (ANOVA) were conducted using JMP software version 9.0 (SAS Institute Inc., Cary, NC, USA) and mean separations performed using the LSD test (*p* < 0.05).

## Results and Discussion

### Effect of Wounding Stress, Phytohormone Treatment, and Storage Time on the Accumulation of Phenolic Compounds

**FIGURE 1:**
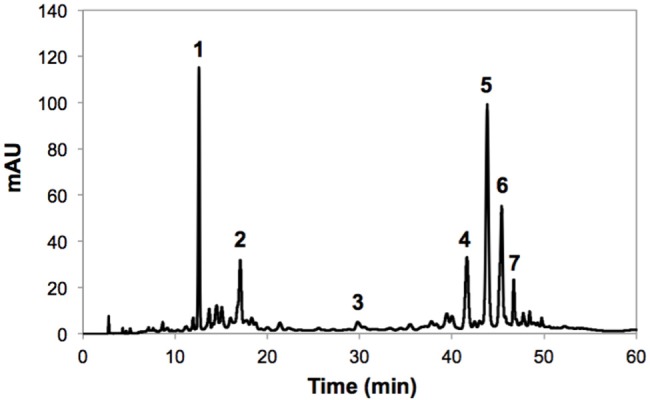
**Typical HPLC-DAD chromatogram (shown at 320 nm) from methanol extracts of broccoli whole heads before storage.** Tentative identification of peaks was performed as indicated in **Table 1**. Peak assignment: (1) 3-*O*-CQA; (2) 5-*O*-CQA; (3) 1,2-DSG; (4) 1-S-2-FG (5) 1,2,2-TSG; (6) 1,2-DFG; (7) 1,2-DS-2-FG.

**FIGURE 2:**
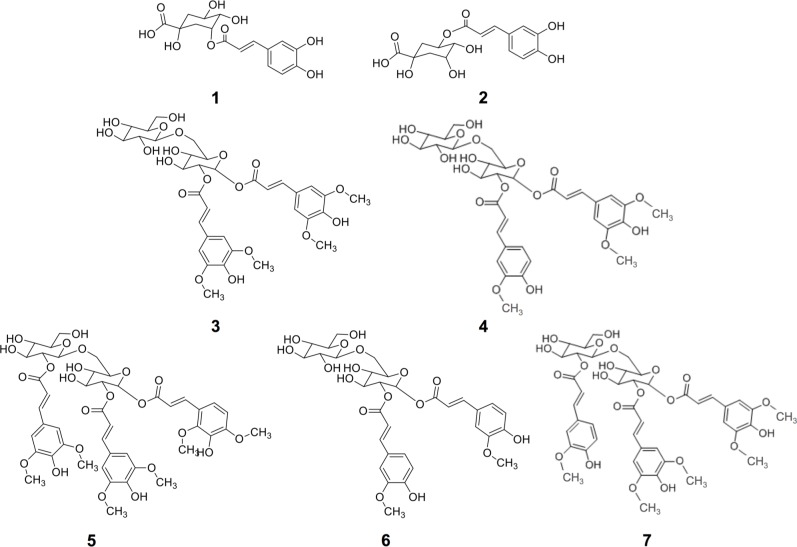
**Chemical structures of phenolic compounds identified in broccoli subjected to wounding stress and exogenous ET and MeJA: 5-*O*-caffeoylquinic acid (3-*O*-CQA, **1**), 5-*O*-caffeoylquinic acid (5-*O*-CQA, **2**), 1,2-disinapoylgentiobiose (1,2-DSG, **3**), 1-sinapoyl-2-ferulolylgentiobiose (1-S-2-FG, **4**), 1,2,2-trisinapoylgentiobiose (1,2,2-TSG, **5**), 1,2-diferulolylgentiobiose (1,2-DFG, **6**), and 1,2-disinapoyl-2-ferulolylgentiobiose (1,2-DS-2-FG, **7**)**.

The identification of individual phenolic compounds present in broccoli treated with or without wounding and phytohormones is shown in **Figure 1** and **Table 1**. The chemical structure of individual phenolic compounds identified are shown in **Figure 2** (compounds **1–7**) and included 3-*O*-CQA (compound **1**), 5-*O*-caffeoylquinic acid (5-*O*-CQA, compound **2**), 1,2-disinapoylgentiobiose (1,2-DSG, compound **3**), 1-sinapoyl-2-ferulolylgentiobiose (1-S-2-FG, compound **4**), 1,2,2-trisinapoylgentiobiose (1,2,2-TSG, compound **5**), 1,2-diferulolylgentiobiose (1,2-DFG, compound **6**), and 1,2-disinapoyl-2-ferulolylgentiobiose (1,2-DS-2-FG, compound **7**). Phenolic compounds identified in broccoli samples herein evaluated (**Figures 1** and **2**; **Table 1**) agree with a previous report (Vallejo et al., 2003). However, in the present study 1,2,2-TSG was identified as the major phenolic compound in broccoli instead of 3-*O*-CQA. The observed differences in phenolic profiles may be due to different cultivation conditions and genetic variation, although extraction parameters are also likely to influence the quantification of phytochemicals (Luthria, 2012).

**Table 1:** Tentative identification of individual phenolic compounds in broccoli.

Peak number (retention time, min)	λmax (nm)	Tentative identification	[M-H]^-^ (m/z)
1 (12.8)	295, 332	3-*O*-CQA^a,b^	353
2 (17.2)	295, 332	5-*O*-CQA^b^	353
3 (30.1)	328	1,2-DSG^b^	753
4 (44.3)	295, 328	1-S-2-FG^b^	723
5 (45.8)	328	1,2,2-TSG^b^	959
6 (47.1)	290,328	1,2-DFG^b^	693
7 (48.2)	295, 328	1,2-DS-2-FG^b^	929


*Identification was obtained by HPLC-DAD-ESI-MS^n^.*

*^a^Identified based on their spectra characteristics and their mass-to-charge ratio as compared with authentic standards. ^b^Identified based on their spectra characteristics and their mass-to-charge ratio as compared with a previous report (Vallejo et al., 2003). 3-*O*-caffeoylquinic acid (3-*O*-CQA); 5-*O*-caffeoylquinic acid (5-*O*-CQA); 1,2-disinapoylgentiobiose (1,2-DSG); 1-sinapoyl-2-ferulolylgentiobiose (1-S-2-FG); 1,2,2-trisinapoylgentiobiose (1,2,2-TSG); 1,2-diferulolylgentiobiose (1,2-DFG); 1,2-disinapoyl-2-ferulolylgentiobiose (1,2-DS-2-FG).*

The phenolic profile of broccoli was affected by the treatments applied (**Table 2**). In the specific case of broccoli heads, storage at 20°C for 24 h resulted on higher levels of phenolics, which were reflected on ∼33, ∼30, and 46% higher levels of 1,2,2-TSG, 1,2-DFG, and 1,2-DS-2-FG, respectively, while the content of 3-*O*-CQA, 5-*O*-CQA, 1,2-DSG, and 1-S-2-FG was not affected by storage. Similar observations have been previously reported in the literature. For instance, Starzyńska et al. (2003) reported ∼22% higher levels of total phenols in broccoli heads stored for 24 h at 20°C. Similarly, Costa et al. (2006) showed that broccoli heads stored for 2 days at 20°C had ∼36% more total phenols than samples before storage. These increments in phenolics observed after storage of broccoli heads could be attributed to a transient increase in the activity of phenylalanine ammonia-lyase (PAL), a key enzyme involved in the biosynthesis of phenolic compounds, as has been previously reported during the first 12 h of storage of broccoli heads stored at 20°C (Porras-Baclayon et al., 2007).

**Table 2:** Concentration of individual phenolic compounds in whole and wounded broccoli tissue treated with and without ethylene (ET) and methyl jasmonate (MeJA) and stored for 24 h at 20°C.

Treatment	Sample	Individual phenolic concentration (mg/kg)^1,2,3^						
		
		3-*O*-CQA	5-*O*-CQA	1,2-DSG	1-S-2-FG	1,2,2-TSG	1,2-DFG	1,2-DS-2-FG
Control (0 h)	Whole	266.57 ± 8.56 bc	124.07 ± 2.99 a	20.19 ± 2.37 c	164.04 ± 6.97 ab	432.18 ± 20.75 bcde	229.99 ± 20.75 bcd	45.36 ± 3.28 ef
Air Control (24 h)	Whole	270.40 ± 5.65 bc	121.19 ± 2.86 a	29.24 ± 2.50 c	199.62 ± 7.48 a	575.40 ± 41.76 a	300.26 ± 43.75 a	66.54 ± 9.35 abc
	Floret	326.75 ± 6.04 a	109.35 ± 2.58 a	57.65 ± 3.14 a	164.85 ± 50.73 ab	487.10 ± 6.10 b	200.01 ± 11.74 cde	74.89 ± 4.82 a
	Wounded floret	242.20 ± 5.70 cd	106.46 ± 13.58 a	24.32 ± 8.25 c	49.68 ± 36.35 d	395.62 ± 26.75 de	220.40 ± 11.35 bcde	53.25 ± 1.10 de
Ethylene (24 h)	Whole	281.52 ± 11.87 b	124.75 ± 8.84 a	16.44 ± 3.21 c	171.31 ± 12.47 ab	475.72 ± 31.23 bc	237.45 ± 37.86 abcd	61.20 ± 3.63 bcd
	Floret	287.37 ± 7.53 b	78.21 ± 6.71 b	50.56 ± 3.34 b	157.22 ± 5.60 bc	492.02 ± 23.86 b	185.01 ± 12.42 cde	62.81 ± 4.36 bcd
	Wounded floret	252.35 ± 19.80 bcd	119.94 ± 9.18 a	37.11 ± 8.08 c	151.08 ± 6.22 bc	445.21 ± 25.59 bcd	273.24 ± 31.16 ab	69.67 ± 9.22 ab
Methyl Jasmonate (24 h)	Whole	215.48 ± 8.60 d	119.90 ± 1.49 a	14.32 ± 1.02 c	154.65 ± 9.43 bc	430.58 ± 14.63 bcde	169.71 ± 9.80 de	38.33 ± 7.72 f
	Floret	282.52 ± 15.45 b	56.18 ± 2.71 c	50.64 ± 3.19 b	123.85 ± 8.86 c	375.17 ± 22.61 de	152.17 ± 5.49 e	57.19 ± 4.35 cd
	Wounded floret	242.81 ± 24.64 cd	114.27 ± 3.86 a	18.46 ± 0.20 c	139.54 ± 3.59 bc	409.66 ± 21.39 cde	240.13 ± 20.55 abc	60.86 ± 10.24 bcd


*^1^Concentrations are reported as 3-*O*-CQA equivalents. All compounds were quantified at 320 nm. ^2^Values represent the mean of three replicates ± standard error of the mean. ^3^Different letters in the same column indicate statistical difference by the LSD test (*p* < 0.05). 3-*O*-caffeoylquinic acid (3-*O*-CQA); 5-*O*-caffeoylquinic acid (5-*O*-CQA); 1,2-disinapoylgentiobiose (1,2-DSG); 1-sinapoyl-2-ferulolylgentiobiose (1-S-2-FG); 1,2,2-trisinapoylgentiobiose (1,2,2-TSG); 1,2-diferulolylgentiobiose (1,2-DFG); 1,2-disinapoyl-2-ferulolylgentiobiose (1,2-DS-2-FG).*

On the other hand, when exogenous ET was applied to whole heads, the accumulation of 1,2,2-TSG was inhibited, whereas MeJA induced a decrease by ∼19% in the content of 3-*O*-CQA as compared to time 0 h control samples, and repressed the accumulation of 1-S-2-FG, 1,2,2-TSG, 1,2-DFG, and 1,2-DS-2-FG (**Table 2**). Although the effects of postharvest treatments with ET and MeJA on the accumulation of phenols in broccoli heads has not been previously studied, Heredia and Cisneros-Zevallos (2009a) reported that the postharvest exposure of whole fresh produce, such as asparagus, potatoes, apples, peaches, strawberries, and grapes, to ET and MeJA had no effect on the total phenolic content of each crop after four days of storage at 20°C. Additionally, other reports by the same authors showed that the concentration of total phenolics in whole carrots treated with ET or MeJA remained unchanged throughout 12 days of storage at 15°C (Heredia and Cisneros-Zevallos, 2009b). However, in both cases, whole tissues stored under air control conditions did not show an enhanced content of total phenolic compounds. This particular difference between the results obtained herein and previous reports may be due to a variation among crops in the response to the stress caused by storage conditions (time and temperature). Moreover, Yuan et al. (2010) reported that the treatment of broccoli florets with 1-methylcyclopropene (1-MCP), an inhibitor of ET action, induced an increase on the activity of the enzyme superoxide dismutase, which produces hydrogen peroxide (H_2_O_2_), a signaling molecule involved on the activation of PAL gene expression and enzymatic activity (Jacobo-Velázquez et al., 2011). Besides, Jacobo-Velázquez et al. (2015) reported a repression in ROS levels as a response to ET treatment in shredded carrots. Therefore, it is likely that broccoli heads treated with exogenous ET could present lower levels of ROS, leading to lower activation of PAL and lower accumulation of total phenolics.

Regarding the concentration of phenolic compounds in broccoli florets before and after storage under air control conditions, the content of 3*-O*-CQA, 1,2-DSG, and 1,2-DS-2-FG showed an increase of ∼22, ∼185, and ∼65%, respectively, as compared to time 0 h control samples, whereas the concentration of 5*-O*-CQA, 1,2-DFG, 1-S-2-FG, and 1,2,2-TSG remained unaltered (**Table 2**). In agreement with this observation, a previous report showed that the total phenolic content remained unchanged during the first twelve days of storage of broccoli florets stored at 5°C (Amodio et al., 2014). Likewise, Du et al. (2014) reported that broccoli florets stored for three days at 15°C showed a slight but significant increase in total soluble phenols (1.1-fold) due to wounding. Chlorogenic acid (3-*O*-CQA) is one of the principal precursors of lignin, whereas 1,2-DSG, and 1,2-DS-2-FG are glycosides of sinapic and ferulic acid, which aglycones are also utilized for the biosynthesis of coniferyl alcohols, precursors of lignin (Vanholme et al., 2010; Torres-Contreras et al., 2014a,b). Therefore, the higher levels of 3-*O*-CQA, 1,2-DSG, and 1,2-DS-2-FG observed after storage of broccoli florets under air control conditions may be related with the wound-induced activation of the phenylpropanoid metabolism, which is required for the biosynthesis of lignin that in wounded plant tissue serves as a water impermeable barrier that prevent excessive water loss (Whetten and Sederoff, 1995; Boerjan et al., 2003; Becerra-Moreno et al., 2015).

The application of ET to broccoli florets during storage inhibited the accumulation of 3-*O*-CQA, and 1,2-DS-2-FG, whereas the accumulation of 1,2-DSG was decreased by ∼12% as compared to air control florets (**Table 2**). Likewise, ET treated broccoli florets showed ∼36% lower levels of 5*-O*-CQA as compared with samples before storage (**Table 2**). It has been reported that 1-MCP induces the downregulation in the expression of genes related with lignin biosynthesis in *Brassica chinensis*, while ET upregulates them (Zhang et al., 2010). Therefore, the less intense accumulation of 1,2-DSG and 1,2-DS-2-FG, as well as the impeded accumulation of 3-*O*-CQA and lower levels of 5-*O*-CQA observed in ET treated florets, suggests an increased rate of lignin biosynthesis induced by ET. Florets treated with MeJA showed a more moderate accumulation of 1,2-DSG and 1,2-DS-2-FG and a decrease of 5-*O*-CQA as compared with the air control (**Table 2**). Additionally, lower concentrations of 1-S-2-FG and 1,2-DFG were observed. Previous reports showed that JA downregulates genes involved in the biosynthesis of phenolic compounds, such as *PAL* and *4-coumarate-CoA ligase* (*4CL*), as well as genes involved in the biosynthesis of lignin, such as the *caffeoyl-CoA 3-O-methyltransferase* (*CCoAOMT*) gene (Jacobo-Velázquez et al., 2015). Therefore, unlike ET-treated samples, the observed MeJA induced decrease and repression of accumulation of individual phenolic compounds are likely due to a downregulation of genes involved in the secondary metabolic pathways leading to the biosynthesis of phenolic compounds.

The application of additional wounding stress to broccoli florets (florets cut into four even pieces) induced a decrease in concentration of 1-S-2-FG by ∼69% after 24 h of storage, whereas the concentration of the other phenolics remained unchanged (**Table 2**). As earlier described, the decrement in phenolics induced by wounding could be attributed to their conversion into lignin, which is needed to prevent excessive water loss in wounded plants (Whetten and Sederoff, 1995; Boerjan et al., 2003; Becerra-Moreno et al., 2015). Therefore, it is likely that wounded broccoli florets experienced a higher rate of lignification than the rate of phenolics biosythesis, and thus, lower levels of 1-S-2-FG were detected. The phenolic compounds identified in broccoli have a glycosylated structure in which two or three simple phenolics may be attached (compounds **3–7**, **Figure 2**). In the case of wounded florets stored under air control conditions, the main phenolic compound affected by wounding (1-S-2-FG) has one sinapic acid and one ferulic acid attached to the carbohydrate moiety. Therefore, it could be hypothesized that phenolic glycosides with a lower number of simple phenolics in their structures are more prone to be hydrolyzed and used as lignin building blocks.

ET applied to wounded-florets resulted on ∼53% higher levels of 1,2-DS-2-FG as compared to samples before storage and ∼30% higher content than air control wounded florets. Furthermore, the application of ET in wounded florets impeded the decrease in concentration observed in 1-S-2-FG after 24 h of storage of wounded florets (**Table 2**). As described earlier, previous reports indicates that ET activates the expression of genes related with phenolics and lignin biosynthesis in wounded plants (Jacobo-Velázquez et al., 2015). In the specific case of wounded carrots, the application of exogenous ET to the tissue increased PAL activity and phenolics accumulation (Heredia and Cisneros-Zevallos, 2009b). Therefore, it is likely that in wounded broccoli florets, exogenous ET increased the biosynthesis rate of phenolic compounds as compared to the air control, and since phenolic biosynthesis increased, the balance between phenolic production and utilization for lignin biosynthesis resulted in no change in total phenolic content.

The application of MeJA to wounded florets only affected the concentration of 1,2-DS-2-FG as compared to air control samples, where exogenous MeJA impeded the wound-induced decrease in concentration observed after storage. These results are in agreement with a previous report, where the application of MeJA to wounded carrots stored for 12 day at 15°C did not induce a significant increase in the concentration of total phenolics (Heredia and Cisneros-Zevallos, 2009b). Additionally, pre-harvest studies have shown that the concentration of phenolic compounds in broccoli is not affected in response to treatment with MeJA. For instance, Barrientos-Carvacho et al. (2014) reported that the treatment of broccoli sprouts with three different concentrations of MeJA (10, 50, 90 μM) induced a decrease in total phenolic compounds. Similarly, a study by Ku and Juvik (2013) showed that the application of MeJA (250 μM) to aerial tissues of broccoli 4 days prior to harvest had no effect on the concentration of phenolic compounds in broccoli florets. These observations may be due to the downregulation that JA exerts on genes related with phenolics and lignin biosynthesis in wounded plants (Jacobo-Velázquez et al., 2015). Therefore, results presented herein indicate that MeJA is not an elicitor for the phenylpropanoid pathway in broccoli, however, since 1-S-2-FG content was increased in wounded-florets treated with MeJA as compared with the air control, it is likely that MeJA selectively induce the accumulation of 1-S-2-FG in wounded tissue (**Table 2**).

Given their health-promoting properties, the production of phenolic compounds in broccoli would be of great interest for the pharmaceutical and dietary supplements industry. For instance, 3-*O*-caffeoylquinic acid has been associated with the reduction of the risk of developing cardiovascular diseases, type II diabetes, and neurodegenerative diseases (Farah et al., 2008). Furthermore, ferulic acid and sinapic acid, the phenolic aglycones of 1,2-DSG, 1-S-2-FG, 1,2,2-TSG, 1,2-DFG, and 1,2-DS-2-FG, are important antioxidants that inhibit the peroxidation of LDL, helping to prevent the progression of atherosclerosis (Natella et al., 1999).

### Effect of Wounding Stress, Phytohormone Treatment, and Storage Time on the Accumulation of Glucosinolates

**FIGURE 3:**
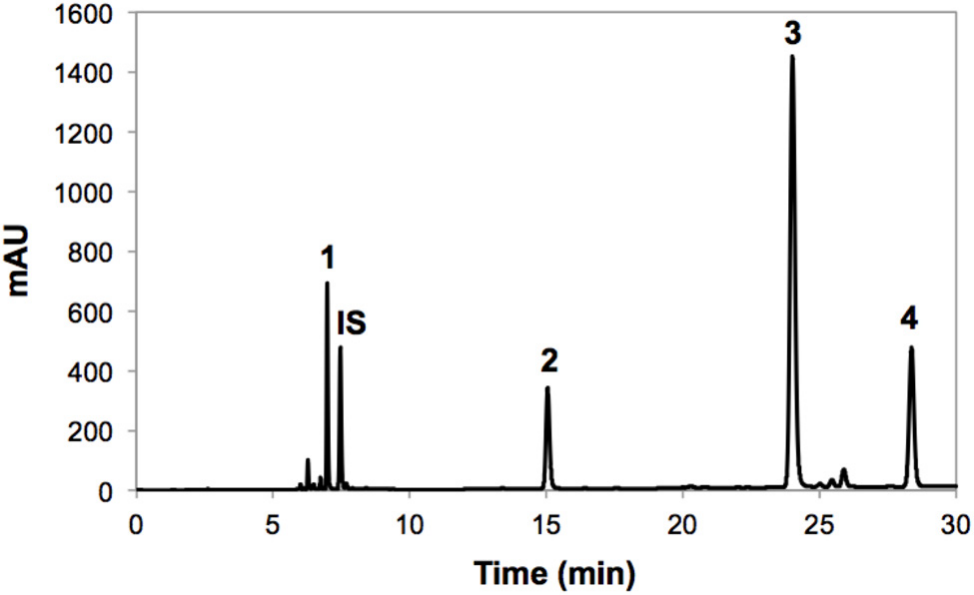
**Typical HPLC-DAD chromatogram (shown at 227 nm) from methanol/water (70/30, v/v) extracts of broccoli whole heads before storage**. Tentative identification of peaks was performed as indicated in **Table 3**. Peak assignment: (1) Glucoraphanin; (2) 4-hydroxyglucobrassicin; (3) Glucobrassicin; (4) Neoglucobrassicin; (IS) Internal standard (sinigrin).

**FIGURE 4:**
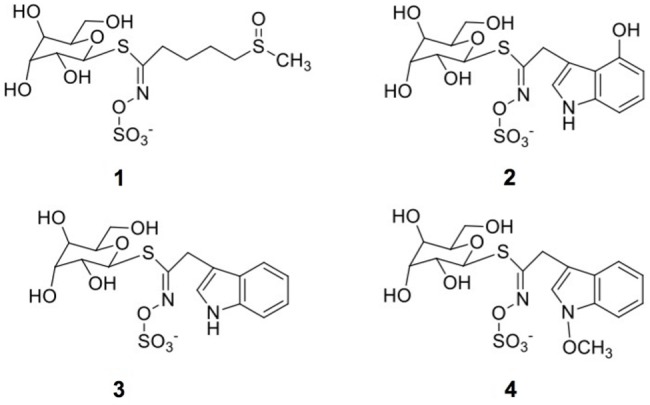
**Chemical structures of glucosinolates identified in broccoli subjected to wounding stress and exogenous ET and MeJA: glucoraphanin (**1**), 4-hydroxyglucobrassicin (**2**), glucobrassicin (**3**), neoglucobrassicin (**4**)**.

**FIGURE 5:**
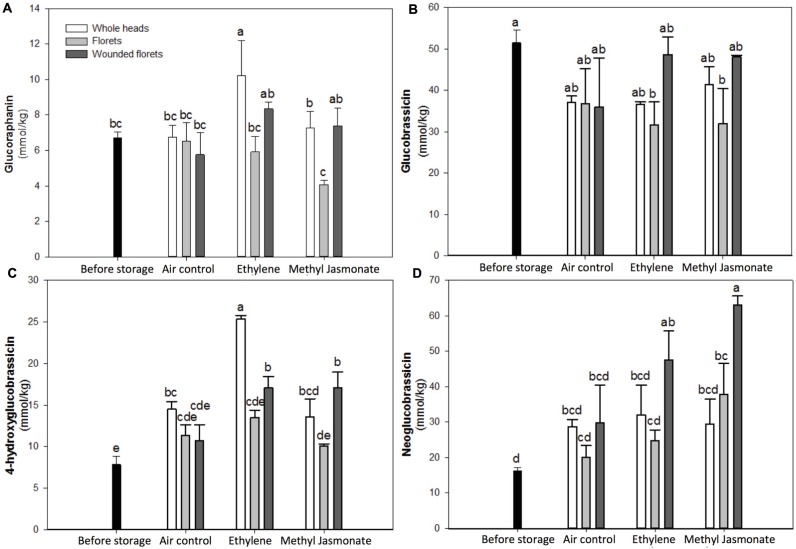
**Effect of wounding and exogenous ET and MeJA on the concentration of glucoraphanin **(A)**, glucobrassicin **(B)**, 4-hydroxyglucobrassicin **(C)**, and neoglucobrassicin **(D)** of broccoli stored for 24 h at 20°C**. Data represent the mean of three repetitions and their standard errors. Data reported for samples before storage represent values of time 0 h whole heads. Bars with different letters indicate statistical difference by the LSD test (*p* < 0.05).

The identification of individual glucosinolates present in broccoli treated with or without wounding and phytohormones is shown in **Figure 3** and **Table 3**. The chemical structure of individual glucosinolates identified is shown in **Figure 4** (compounds **1–4**) and includes one aliphatic glucosinolate (glucoraphanin, compound **1**), and three indolic glucosinolates (4-hydroxyglucobrassicin, compound **2**; glucobrassicin, compound **3**; and neoglucobrassicin, compound **4**). Likewise, their concentration in broccoli treated with or without wounding and phyhormones is shown in **Figure 5**.

**Table 3:** Identification of individual glucosinolates in broccoli.

Peak number (retention time, min)	λmax (nm)	Tentative identification	[M-H]^-^ (m/z)
1 (7.1)	222	Glucoraphanin^a,b,c^	380
2 (14.8)	221	4-hydroxyglucobrassicin^b^	–
3 (23.1)	222, 280	Glucobrassicin^b,c^	391
4 (27.5)	222	Neoglucobrassicin^b,c^	421


*Identification was obtained by HPLC-PDA-ESI-MS^n^.*

*^a^Identified based on their spectra characteristics and their mass-to-charge ratio as compared with authentic standards. ^b^Identified based on their spectra characteristics and order of elution as compared with a previous report (Hansen et al., 1996; Vallejo et al., 2003; Barbieri et al., 2008; Miglio et al., 2008). ^c^Identified based on their spectra characteristics and their mass-to-charge ratio as compared with a previous report (Vallejo et al., 2003).*

Whole heads stored for 24 h at 20°C showed an increase of ∼84% in the content of 4-hydroxyglucobrassicin as compared to the control (time 0 h samples), whereas the content of the other three individual glucosinolates remained unchanged (**Figure 5**). When whole broccoli heads were treated with ET, the concentration of glucoraphanin and 4-hydroxyglucobrassicin increased by ∼52 and ∼223%, respectively, as compared with the control (**Figures 5A,C**). These results are in agreement with a previous report, where the expression levels of genes related with glucosinolate biosynthesis strongly correlated with endogenous ET production in broccoli (Ku et al., 2013b). In the indolic glucosinolate biosynthetic pathway, glucobrassicin is synthesized by sulfotransferases 16 and 18 (SOT16 and SOT18), and then glucobrassicin is converted into neoglucobrassicin and 4-hydroxyglucobrassicin by the subfamily of CYP81F genes by methylation and hydroxylation reactions, respectively. Therefore, these observations suggest that, in the specific case of broccoli whole heads, the hydroxylation of glucobrassicin was favored by postharvest storage and ET treatments.

The application of MeJA in whole broccoli heads did not induce additional accumulation of glucosinolates as compared to the air control (**Figure 5**). A previous report where MeJA was applied four days before harvest of broccoli reported an increase in the expression levels of hydrolytic enzymes (myrosinase), which converts glucosinolates into isothiocyanates (Ku et al., 2013b). This suggests that MeJA also acts as a signal that leads to a higher myrosinase activity in whole heads, making it more available for glucosinolate hydrolysis, therefore, it is likely that the glucoraphanin and 4-hydroxyglucobrassicin produced by storage conditions are being hydrolyzed into isothiocyanates, and thus, no accumulation of any individual glucosinolate was observed (**Figure 5**).

Florets stored for 24 h at 20°C under air control conditions did not show significant difference in the glucosinolate profiles as compared with the control (time 0 h samples, **Figure 5**). Treating broccoli florets with ET or MeJA did not affect the concentration of individual glucosinolates as compared with air control samples. The application of additional wounding stress to florets (wounded florets) did not affect the glucosinolate profile of broccoli when stored under air control conditions. However, when wounded florets were treated with ET, the concentration of neoglucobrassicin and 4-hydroxyglucobrassicin was enhanced by ∼193 and ∼117% as compared to the control (time 0 h samples), whereas the concentration of the other individual glucosinolates remained unaltered as compared with the air control. Likewise, MeJA treatments induced significant increments in the levels of neoglucobrassicin and 4-hydroxyglucobrassicin, where their concentrations were increased by ∼286 and ∼117% as compared with time 0 h samples. Results suggest that MeJA and ET induced the activation of CYP81F genes involved on glucobrassicin hydroxylation and methoxylation, forming 4-hydroxyglucobrassicin and neoglucobrassicin, respectively. These results are in agreement with studies in *Arabidopsis* indicating that genes related to indolic glucosinolate biosynthesis are more susceptible to be induced by exogenous phytohormones rather than those playing a role in aliphatic glucosinolate biosynthesis, although the latter may also be up-regulated (Mikkelsen et al., 2003). Interestingly, in the present study, it was shown that to accumulate indolic glucosinolates (neoglucobrassicin and 4-hydroxyglucobrassicin) the combination of wounding stress with MeJA or ET was required, whereas for the accumulation of aliphatic glucosinolate (glucoraphanin) the sole application of ET on broccoli heads was sufficient (**Figure 5**). The unchanged levels of glucoraphanin in response to exogenous phytohormones in broccoli treated with wounding stress (florets and wounded florets) may be due to a selective wound-induced activation of an aliphatic-specific myrosinase.

The accumulated glucosinolates in stressed broccoli have diverse industrial applications. For instance, in the pharmaceutical and dietary supplements industry, glucoraphanin has gained interest in the last few years due to the anticarcinogenic properties of its hydrolysis product, sulforaphane (Fahey and Talalay, 1999). Additionally, glucosinolates can also be used as insecticides to protect horticultural crops, as reported by El Sayed et al. (1996), who showed an inhibiting effect of glucobrassicin and its hydrolysis product, indol-3-ylmethylisothiocyanate, on *Schistocerca gregaria*, an insect that threatens crop production mainly in Africa, Middle East, and Asia.

## Conclusion

Results presented herein showed that simple postharvest treatments such as wounding applied alone or in combination with exogenous phytohoromnes (ET and MeJA) can be used as an effective emerging technology that allows the accumulation of specific glucosinolate and phenolic compounds in broccoli. For instance if the accumulation of specific phenolic compounds such as 2,2-TSG, 1,2-DFG, and 1,2-DS-2- FG are desired whole broccoli heads can be stored for 24 h at 20°C. Furthermore, for the accumulation of 3*-O*-CQA, 1,2-DSG, and 1,2-DS-2-FG, broccoli florets should be stored under the same condition. However, when broccoli was treated with ET or MeJA the accumulation of these individual phenolics was impeded. On the other hand, if the accumulation of glucoraphanin and 4-hydroxyglucobrassicin is desirable, whole broccoli heads should be treated with exogenous ET for 24 h. Likewise, for the accumulation of neoglucobrassicin wounded broccoli florets should be treated with exogenous ET and MeJA during 24 h at 20°C. This particular observation suggests a complex cross-talk between wounding and the applied phytoregulators acting on the metabolism of glucosinolates. Despite that quality changes in broccoli were not a factor evaluated in this study, the visual quality of broccoli heads was not affected within the 24-h period of storage, suggesting that it could be used as a functional food. However, further studies should be performed to validate consumers acceptability and microbial safety of the tissue. Additionally, the stressed broccoli tissue with increased levels of bioactive molecules could be subjected to downstream processing in order to extract and purify the bioactive compounds for their subsequent use on the dietary supplements, agrochemical and cosmetics markets.

## Author Contributions

DV-G, and DJ-V designed experiments. DV-G and VN, carried out experiments. DV-G and VN, processed data. DV-G, LC-Z, and DJ-V, analyzed data and wrote the main text of the manuscript. All authors read and approved the final manuscript.

## Conflict of Interest Statement

The authors declare that the research was conducted in the absence of any commercial or financial relationships that could be construed as a potential conflict of interest.
